# Establishment of a Rapid LAMP Assay for *Aeromonas hydrophila* and Comparison with the Application of qPCR

**DOI:** 10.3390/metabo13070841

**Published:** 2023-07-12

**Authors:** Zihui Gao, Chunhua Yang, Xiaobo Zhang, Bing Hu, Huang Zhang, Zhihong Zhang, Wendong Kuang, Qiuyue Zheng, Jijuan Cao

**Affiliations:** 1 Key Laboratory of Biotechnology and Bioresources Utilization of Ministry of Education, College of Life Science, Dalian Minzu University, Dalian 116600, China; 2 Institute of Biological Resources, Jiangxi Academy of Sciences, Nanchang 330096, China; 3 Guangzhou Double Helix Gene Technology Co., Ltd., Guangzhou 510320, China

**Keywords:** *Aeromonas hydrophila*, LAMP, qPCR

## Abstract

The development of an exceptionally sensitive diagnostic technique for early identification of aquaculture diseases, specifically Aeromonas hydrophila, is essential for efficient management of disease outbreaks at aquaculture locations. In this research, a swift and sensitive diagnostic assay employing Loop-mediated isothermal amplification (LAMP) of Aeromonas hydrophila was devised and compared to the conventional qPCR method documented by Rong Wang. Validation of the diagnostic assay was carried out using actual samples obtained from aquaculture fish. The findings revealed that based on the rapid detection of crude bacterial genomic DNA, the fluorescent LAMP assay possessed a lower limit of detection (LOD) of 0.559 ng/μL (0.315–1.693, 95% CI), while the LOD for qPCR stood at 4.301 ng/μL (2.084–8.876, 95% CI). Both techniques demonstrated outstanding specificity, exhibiting no cross-reactivity with bacteria from the same or closely related genera. A total of 74 fish samples suspected to be infected with the fish disease were gathered, with 26 and 23 samples testing positive for Aeromonas hydrophila via LAMP and qPCR, respectively. The concordance analysis for LAMP and qPCR methods generated a Kappa value of 0.909 (0.778–1.000, 95% CI), signifying a high degree of diagnostic consensus. This study highlights that the LAMP assay eliminates the thermal cycle temperature change process of qPCR, uses lysate to crudely extract bacterial genomic DNA, and can complete the detection within 40 min, rendering it a practical and efficient alternative for monitoring disease outbreaks at aquaculture sites.

## 1. Introduction

*Aeromonas hydrophila*, a facultative anaerobic Gram-negative bacillus [1], represents a widespread aquatic and zoonotic pathogen present in water bodies, diseased fish, food, and animal waste globally [2]. This pathogen contributes to various diseases in aquaculture animals, including motile *Aeromonas septicemia* and red sore disease, which can lead to significant economic losses due to mass mortality [3]. Moreover, *Aeromonas hydrophila* is an emerging enteric and foodborne pathogen causing infectious diseases in humans, such as cholestasis, necrotizing fasciitis, acute gastroenteritis, ascites, and cloacal hemorrhage, thus posing a threat to the quality and safety of aquatic products and human health [4,5,6,7,8]. At present, antibiotic therapy is the primary treatment for *Aeromonas hydrophila* infections, and no effective cure exists. Consequently, early detection of this pathogen is crucial for prevention and control of these infections [9]. Developing a rapid, sensitive, and precise detection method for *Aeromonas hydrophila* is therefore essential to enhancing treatment and control strategies, mitigating the risk of bacterial infection, and safeguarding the safety of fish, as well as human consumption and health.

Traditional culture and biochemical methods are highly accurate for identifying clinical isolates and ATCC strains of *Aeromonas hydrophila*, but they are time-consuming and necessitate skilled technicians, rendering them unsuitable for rapid field testing [10]. Immunological techniques, such as spot blotting [11] and monoclonal antibodies [12], are faster but more expensive and require antibody preparation, limiting their accessibility on farms. PCR-based assays, including multiplex PCR [13], real-time fluorescent PCR [14], gene sequencing [15], and biosensors [16], provide quick and accurate detection of *Aeromonas hydrophila* but mandate specialized equipment and trained personnel, making them impractical for use in resource-limited labs or in the field. Therefore, a portable, cost-effective, sensitive, and efficient method for rapidly detecting *Aeromonas hydrophila* in the field is urgently required.

Loop-mediated isothermal amplification (LAMP) is a groundbreaking nucleic acid amplification technique that offers multiple advantages over traditional PCR-based assays, such as high specificity, sensitivity, rapidity, constant reaction temperature, and visual detection of results [17,18]. This technology has found extensive application in the detection of pathogenic bacteria. For instance, Liu H developed a sensitive and specific method for detecting *Shigella* spp. using LAMP combined with calcein and nucleic acid assay (NADA), achieving a limit of detection of 2 × 10^2^ copies/μL of the recombinant plasmid containing the target gene, which proved to be ten times more sensitive than traditional PCR-based methods [19]. Similarly, Zhou S devised a LAMP-HNB (hydroxy naphthol blue dye) method for the rapid detection of *Aeromonas salmonicida*, demonstrating high specificity, sensitivity, and a detection limit of 3.077 × 10^−6^ ng/μL without any false-positive or false-negative results [20]. Additionally, LAMP has been employed to diagnose homozygous hemorrhagic gill disease in heterozygous crucian carp caused by Cyprinus carpio herpesvirus-2 (CyHV-2) [21]. The Bst polymerase utilized in LAMP amplification is highly tolerant to nucleic acid inhibitors and can be employed for genetic testing in clinical settings [22], as well as for rapid testing of food and environmental samples [23], even in instances of sample contamination or when high-purity nucleic acid samples are unattainable.

This study sought to establish a rapid and sensitive detection method for *Aeromonas hydrophila* using Loop-Mediated Isothermal Amplification (LAMP) technology. Six LAMP primers were designed, targeting the *aerA* gene of *A. hydrophila*. A straightforward and efficient DNA extraction method was devised for implementation in the LAMP assay. To evaluate the performance of the LAMP assay, specificity and sensitivity tests were carried out. Moreover, the efficacy of the LAMP method was validated by contrasting it with the qPCR method documented in the literature, focusing on the detection of field samples. The findings of this research illustrate the potential of LAMP as a valuable instrument for the rapid and precise detection of *A. hydrophila* across various environments.

## 2. Materials and Methods

### 2.1. Strains and Samples

To validate the method and assess its specificity, standard strains and clinical isolates were employed in this study. The strains used, which included *Aeromonas hydrophila* ATCC49140, 5 *Aeromonas hydrophila* clinical isolates, *Aeromonas sobria* ATCC43979, *Aeromonas veronii* CCTM3019, *Aeromonas schubertii* ATCC43700, *Aeromonas veronii* ATCC35624, *Pseudomonas fluorescens* ATCC13525, *Pseudomonas aeruginosa* ATCC27853, *Pseudomonas stutzeri* ATCC8482, *Vibrio parahaemolyticus* ATCC33847, *Vibriovulnificus* ATCC27562, *Edwardsiella tarda* ATCC15947, *Escherichia coli* ATCC25922, *Enterobacter cloacae* CMCC45301, *Proteus mirabilis* CMCC49005, *Serratia marcescens* CMCC41002, *Streptococcus thermophilus* CICC6038, and *Listeria iuanuii* in sheep ATCC19119, were previously authenticated and maintained in the laboratory. Among them, 5 isolates of *Aeromonas hydrophila* were isolated and purified from fish samples infected with fish diseases in our laboratory. They were confirmed as *Aeromonas hydrophila* by biochemical identification and stored in 10% glycerol at −80 °C. in spare. In this study, 74 fish samples suspected to be infected with hemorrhagic lesions were collected and used as clinical validation samples. The 74 experimental samples were taken from various aquaculture enterprises in Dalian, and samples of fish found sick and dead during the breeding process were sent to this laboratory after being frozen. Screening is based on the following clinical symptoms of diseased fish samples: blackened body of diseased fish, hyperemia at the base of fins, punctate or massive hemorrhage on both sides of the fish body, and severe hyperemia around the mouth, gills, fundus, and orbit of the fish.

### 2.2. Pre-Treatment and DNA Extraction of Samples

Under sterile conditions, we sterilized the fish body surface, take 0.1~0.2 g of gill tissue samples, put them into a grinding tube filled with phosphate-buffered saline buffer (PBS), and ground to make about 10% of the tissue. The homogenate was centrifuged at 5000× *g* for 5 min, and the centrifuged precipitate was collected in a 1.5 mL Microtube, and 100 μL of MightyPrep reagent (Code9182, TaKaRa Co., Ltd., Dalian, China) was added and mixed evenly, heated in a water bath at 95 °C for 10 min, cooled to room temperature, and centrifuged at 12,000 rpm for 2 min. The supernatant was directly used as a template, and the supernatant was directly used for qPCR and fluorescent LAMP analysis.

The standard strain of *Aeromonas hydrophila* was inoculated into LB (Luria-Bertani) broth, cultured at 37 °C for 24 h; 1 mL of broth culture was added to 1.5 mL of Microtube, centrifuged at 5000× *g* for 5 min; and the precipitate was collected. We added 100 μL of MightyPrep reagent (Code 9182, TaKaRa Co., Ltd., Dalian, China) to the precipitate, and mixed well. It was heated in a water bath at 95 °C for 10 min, cooled to room temperature, and centrifuged at 12,000 rpm for 2 min, and the supernatant was taken as a template for qPCR and fluorescent LAMP analysis.

### 2.3. LAMP Primer Design and Specificity Comparison

*Aeromonas hydrophila aerA* sequences were acquired from the National Center for Biotechnology Information (NCBI) database, and representative isolates were selected and analyzed for interspecies homology using DNAStar software (DNAStar Inc., Madison, WI, USA). The *aerA* gene sequence served as a template in a blast search, and the highest-scoring results were chosen for method development. Furthermore, gene sequences of other bacteria belonging to the genus *Aeromonas* were retrieved for exclusivity testing. The gene sequences were compared using MEGA 4.0 software. The *aerA* gene sequence (GU229024.1) was employed as a template with which to design LAMP primers via LAMP Primer Explorer 5 software (Eiken EIKEN, Tokyo, Japan), and the secondary structure and potential presence of primer dimers were assessed. TaKaRa (Dalian) Co., Ltd. synthesized the primers and probes, and the specific sequences are presented in Table 1. The primers and Taq Man probes for qPCR (Fw/Rev/probe) were based on a previous study [14].

### 2.4. Real-Time Fluorescent Quantitative PCR (qPCR)

Real-time fluorescent quantitative PCR (qPCR) was conducted using a Flex QuantStudio TM 7 instrument (Thermo Fisher Scientific, Waltham, MA, USA). The reaction mixture (20 μL) consisted of 10 μL of Probe qPCR Mix (2×), 0.4 μL of PCR Forward Primer (10 μM), 0.4 μL of PCR Reverse Primer (10 μM), 0.8 μL of Probe, 0.2 μL of ROX Reference Dye II (50×), 2 μL of DNA template, and 6.2 μL of RNase Free H_2_O. The thermal cycling conditions were set as follows: initial denaturation at 95 °C for 30 s, followed by 40 cycles of denaturation at 95 °C for 5 s, and annealing/extension at 60 °C for 34 s.

### 2.5. LAMP Fluorescence Method

Fluorescent LAMP analysis was performed on a Flex QuantStudio TM7 real-time fluorescence PCR instrument (USA, Thermo Fisher Scientific). The reaction mixture (25 µL) was prepared according to the DNA Amplification Kit (Thermostatic Amplification Method) (051011M, IP20010301, Guangzhou Double Helix Technology Co., Ltd., Guangzhou, China) system. The mixture included 12.5 µL RM-SCI reaction solution (1×), 1 µL of inner primer FIP/BIP (1.6 µM), 1 µL of outer primer F3/B3 (0.2 µM), 1 µL of loop primer LoopF/LoopB (0.8 µM), 1 µL of Bst polymerase, 0.5 µL of fluorescent dye, 2 µL of DNA template, and 3 µL RNase Free H_2_O. The fluorescence amplification curve was monitored on the real-time fluorescence PCR instrument with FAM as the fluorescent group and None as the quenching group. The reaction was preheated at 63 °C for 30 s, and then 63 °C, 15 s and 63 °C, 45 s were reacted 45 times as a cycle. The fluorescence signal was collected at 63 °C for 45 s, and the cycle threshold (Ct) value was recorded.

### 2.6. Specificity Analysis

Genomic DNA was extracted from 22 strains of *Aeromonas hydrophila*, along with other *Aeromonas* spp., *Pseudomonas* spp., and *Vibrio* spp. bacteria in this study. The samples were subsequently analyzed using the fluorescent LAMP system to evaluate cross-reactivity. To ensure the reliability of the results, two replicates were performed for each sample to verify the specificity of the LAMP method.

### 2.7. Amplification Efficiency

A nutrient broth culture (OD 600 of 0.61) of 200 μL of *Aeromonas hydrophila* was utilized to extract genomic DNA with an initial concentration of 1.43 × 10^2^ ng/μL. The DNA was then diluted to four concentration gradients of 1.43 × 10^2^, 1.43 × 10^1^, 1.43 × 10^0^, and 1.43 × 10^−1^ ng/μL. For each concentration, three parallel reactions were set up, and the experiment was repeated three times using both methods. Linear curves were plotted using the logarithmic value of the starting sample DNA concentration as the horizontal coordinate and the amplified Ct value as the vertical coordinate. The linear equations, slopes, linear correlation coefficient (R^2^) values, and PCR reaction efficiency (E) were calculated. Amplification efficiency was compared between the LAMP and qPCR methods.

### 2.8. Sensitivity Testing

Genomic DNA was extracted from nutrient broth cultures (OD 600 of 0.61) of 200 μL of *Aeromonas hydrophila* with an initial concentration of 1.43 × 10^2^ ng/μL and was used as a template for serial dilutions. Dilutions were made to obtain six concentration gradients of 1.43 × 10^2^, 1.43 × 10^1^, 1.43 × 10^0^, 1.43 × 10^−1^, 1.43 × 10^−2^, and 1.43 × 10^−3^ ng/μL. To test sensitivity, three parallel reactions were set up for each concentration, and the experiment was repeated three times using both methods. In order to be closer to the rapid detection process of actual samples, this study used MightyPrep reagent lysate to quickly extract bacterial genomic DNA and obtained crude nucleic acid extraction products as amplification templates by which LAMP and qPCR can explore the sensitivity under the conditions of simple lysis and extraction reagents.

To determine the limit of detection (LOD) for qPCR and LAMP, further dilutions of genomic DNA lysate were prepared to five concentration gradients of 1.435 ng/μL, 0.717 ng/μL, 0.430 ng/μL, 0.143 ng/μL, and 0.071 ng/μL. Twelve subsamples were tested in parallel for each concentration gradient, and the LOD was defined as the lowest concentration at which all 12 subsamples had positive results. The data were expressed as mean ± standard deviation and analyzed using semi-log regression analysis with GraphPad PRISM (GraphPad Inc., La Jolla, CA, USA). Probabilistic regression analysis was performed on replicate test data from the five serial dilutions of the concentration gradient using MedCalc software to calculate the LOD for LAMP and qPCR at the 95% probability level.

### 2.9. Comparison of Actual Sample Detection Based on qPCR, LAMP

Seventy-four fish samples suspected to be infected with hemorrhagic lesions were obtained and used to evaluate the feasibility of the LAMP assay developed in this study for detecting *Aeromonas hydrophila*. Additionally, the previously reported qPCR method was employed for comparative validation, with each sample tested in duplicate. To assess the agreement between the results obtained from the LAMP and qPCR assays, kappa values were calculated using SPSS Statistics 17.0 software. The range of kappa calculation results is −1~1; usually, kappa falls between 0 and 1, and can be divided into five groups to represent different levels of consistency: 0.0~0.20, very low consistency (slight); 0.21~0.40, general consistency (fair); 0.41~0.60, moderate consistency; 0.61~0.80, substantial consistency; and 0.81~1, almost perfect. Among them, the results of positive samples detected by LAMP and qPCR were confirmed by the SN/T 0751-2010 method [24].

## 3. Results

### 3.1. Specificity Analysis

**Figure 1:**
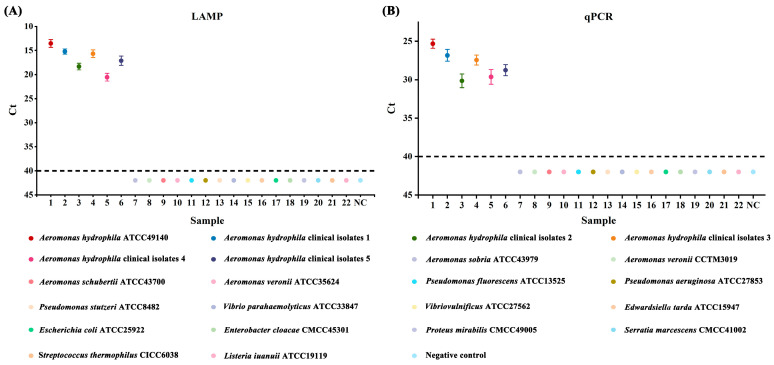
Results of specificity tests for LAMP and qPCR assays. (**A**) *Aeromonas hydrophila* LAMP specificity. (**B**) *Aeromonas hydrophila* qPCR specificity; 1: *Aeromonas hydrophila* ATCC49140; 2–6: *Aeromonas hydrophila* isolates; 7–22: non-*Aeromonas hydrophila* strains; NC negative control.

Genomic DNA obtained from the *Aeromonas hydrophila* ATCC 49140 reference strain, five clinical isolates, and sixteen other strains were subjected to LAMP and qPCR reactions to evaluate their specificity. The number of strain samples was plotted on the *x*-axis, and the amplification Ct values were plotted on the *y*-axis, with a lack of amplification signals indicated by Ct values greater than 40 (as depicted in Figure 1). The results indicated that a positive fluorescence signal was observed only in the presence of *Aeromonas hydrophila* genomic DNA in the reaction mixture. Furthermore, the genomic DNA extracted from other strains did not show any positive amplification signal, indicating the high specificity of both methods.

### 3.2. Amplification Efficiency Analysis

Genomic DNA of *Aeromonas hydrophila* were used as a template at various concentrations (initial concentration 1.43 × 10^2^ ng/μL) for LAMP and qPCR amplification. The logarithmic value of the template DNA concentration was used as the horizontal coordinate, while the Ct value was used as the vertical coordinate to plot the linear curves for both methods using Origin 2022 software.

**Figure 2:**
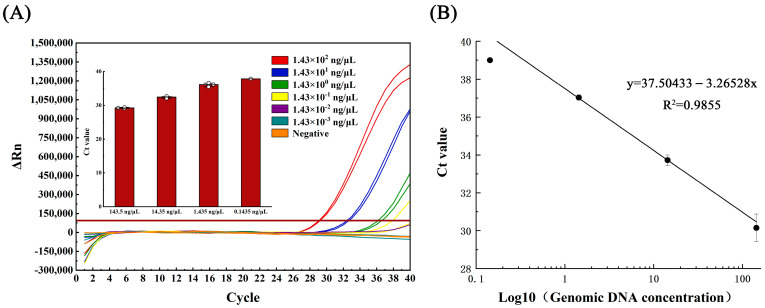
(**A**) Amplification curve and (**B**) linearity curve of qPCR test for gradient dilution of *Aeromonas hydrophila* DNA.

The standard curve equations, correlation coefficients, and amplification efficiency were determined for qPCR and LAMP methods using the extracted genomic DNA of *Aeromonas hydrophila* at gradient dilution. The qPCR linear curve was generated using the equation y = 37.504 − 3.265 lg X, where X is the logarithmic value of the template DNA concentration, with a correlation coefficient (R^2^) of 0.985 and an amplification efficiency of 102.43%, calculated via the amplification efficiency equation E = 10(1/slope) − 1 (refer to Figure 2B).

**Figure 3:**
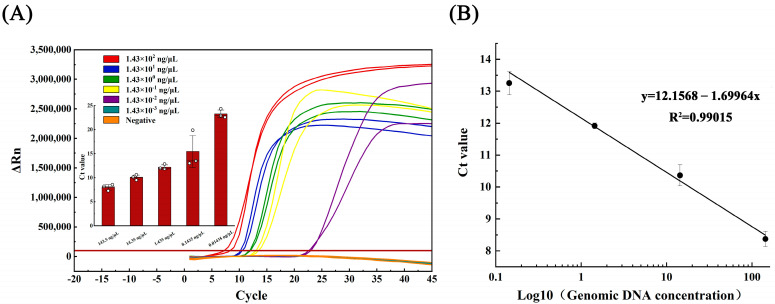
(**A**) Amplification curve and (**B**) linearity curve of LAMP test for gradient dilution of Aeromonas hydrophila DNA.

Similarly, the LAMP linear curve was plotted using the equation y = 12.156 − 1.699 lgX, with a correlation coefficient (R^2^) of 0.990 and an amplification efficiency of 287.78%, calculated via the amplification efficiency equation E = 10(−1/slope) − 1 (refer to Figure 3B).

### 3.3. Sensitivity Testing

The genomic DNA of *Aeromonas hydrophila* (initial concentration 1.43 × 10^2^ ng/μL), which crudely exacted nucleic acid from the lysate, was subjected to serial dilutions, generating five concentration gradients of 1.43 × 10^1^, 1.43 × 10^0^, 1.43 × 10^−1^, 1.43 × 10^−2^, and 1.43 × 10^−3^ ng/μL. In the qPCR assay, detection occurred at 1.43 × 10^−1^ ng/μL, with only one of the three parallel samples yielding positive results (Figure 2A). Conversely, the LAMP assay detection occurred at 1.43 × 10^−2^ ng/μL, with all three parallel samples producing positive results (Figure 3A).

**Figure 4:**
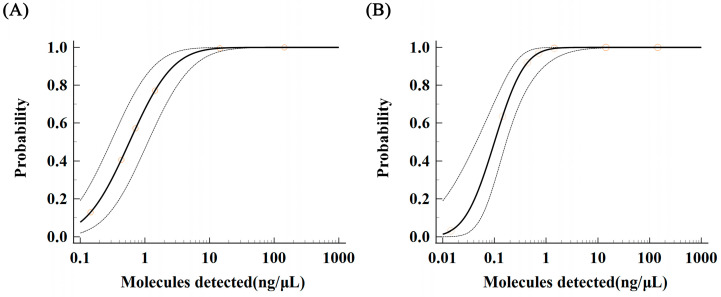
LOD and confidence interval analysis for the detection of *Aeromonas hydrophila* by qPCR and LAMP. (**A**) LOD probability regression analysis of qPCR. (**B**) LOD probability regression analysis of fluorescent LAMP. Note: The solid line in the figure represents the probability regression curve of the number of times that can be detected by 12 repeated detections for each serial dilution concentration at the 95% probability level; the dotted line represents the range of confidence interval; the circle represents the 12 times of each serial dilution concentration gradient test The probability of the number of times that repeated detection can detect.

Samples with genomic DNA at various concentration gradients, ranging from 1.435 ng/μL to 0.071 ng/μL, were evaluated in this study. For each concentration gradient, 12 subsamples were tested in parallel to ensure accuracy. The study data were analyzed using MedCalc software to calculate the low limit of detection (LOD) for both LAMP and qPCR methods at a 95% probability level. For the qPCR method, the LOD was determined to be 4.301 ng/μL with a 95% confidence interval of 2.084–8.876 ng/μL for the detection of *Aeromonas hydrophila* DNA (Figure 4A). In contrast, the LOD of the LAMP method was calculated to be 0.559 ng/μL with a 95% confidence interval of 0.315–1.693 ng/μL for the detection of *Aeromonas hydrophila* DNA (Figure 4B).

### 3.4. Comparative Analysis of qPCR and LAMP Assays for the Detection of Real Samples

To assess the practical utility of the qPCR and LAMP methods for detecting *Aeromonas hydrophila* in real samples, genomic DNA extracted from 74 fish samples suspected to be infected with hemorrhagic lesions was detected. Two parallel experiments were performed on each sample, and the detection results of different methods were compared. Using the LAMP method, 26 samples were identified as positive for *Aeromonas hydrophila* DNA with Ct values ranging from 8.018 to 15.511, while the remaining 48 samples were negative. The literature-reported qPCR method identified 23 samples as positive, while the remaining 51 samples were negative. The standard method SN/T 0751-2010 [25] was used to confirm the 74 samples. Notably, three samples that tested negative by the qPCR method were positive by LAMP, and the positive result was confirmed by the SN/T 0751-2010 [25] method to be consistent with the LAMP test result.

**Figure 5:**
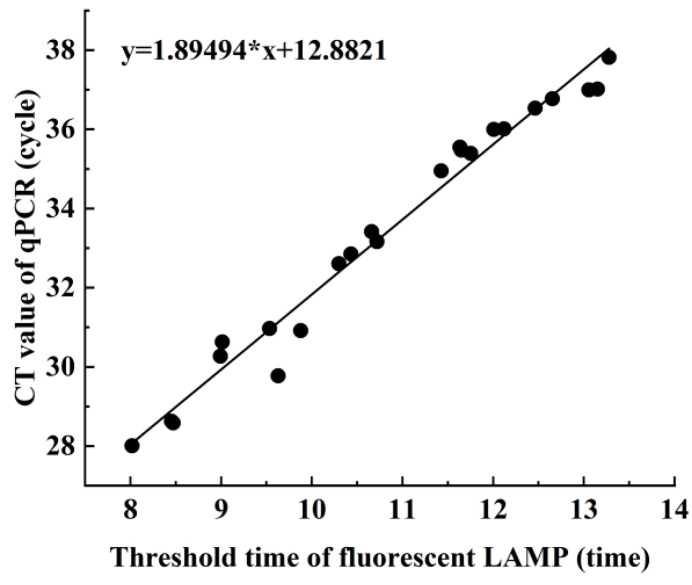
Comparison of LAMP and qPCR in detecting *Aeromonas hydrophila* in clinical samples (*n* = 74). *X*-axis: LAMP detection Ct value; *Y*-axis: qPCR detection of Ct value.

Linear correlation analysis (Figure 5) was performed to investigate the relationship between the Ct value of qPCR and the threshold time for fluorescent LAMP during the assay. The results showed a positive linear correlation, indicating that as the Ct value of qPCR increased, the threshold time for fluorescent LAMP increased as well. Furthermore, a concordance analysis of the actual sample assay was conducted, and the kappa value between the LAMP and qPCR methods was found to be 0.909 (95% CI: 0.778 to 1.000), indicating a high degree of diagnostic concordance between the two methods for the detection of *Aeromonas hydrophila* in the actual sample.

## 4. Discussion

*Aeromonas hydrophila* infection is a continuous cause of fish disease and economic loss in the aquaculture industry. As a potential foodborne pathogen, it also poses a serious threat to food safety and human health. Therefore, early diagnosis, disease monitoring, and prevention of fish infection are crucial. Studies have shown that hemolysin, cytotoxicity, enterotoxin, aerolysin, cell excitatory enterotoxin, and extracellular protease are considered to be the main pathogenic virulence genes of *Aeromonas hydrophila*, and these toxins have strong toxicity, which is one of the direct causes of ulcers, bleeding, and necrosis in body tissues. Zhu Daling reported that there is a correlation between the *aer* and *ahp* genes and the virulence of *Aeromonas hydrophila*; the strains carrying the *aer* and *ahp* genes may be virulent strains; and the strains carrying these virulence genes can cause disease outbreaks Popularity [26]. Moreover, in the process of subculture of *Aeromonas hydrophila*, the strain is prone to mutation, and repeated transplantation and subculture will reduce the pathogenicity of the strain, but the carrying rate of the aerolysin gene *aerA* has not changed, and its genetic stability higher [24].

Therefore, we developed a LAMP method to detect *Aeromonas hydrophila* using the aerolysin gene. We designed selected the position of the conserved region 719–903 of the *Aeromonas hydrophila* aerolysin gene from Genbank and two sets of primers were selected for LAMP amplification. After primer screening and optimization, a set of primers with high amplification efficiency was selected. In order to further improve the specificity and sensitivity of the method, a temperature gradient was set to optimize the isothermal amplification temperature, and a temperature gradient of 59–65 °C was selected; at the same time, the isothermal amplification reaction time was optimized, and the reaction time was set to 20 min, 30 min, and 40 min, respectively. Finally, the optimized LAMP reaction conditions were obtained, and the isothermal amplification was completed at 63 °C for 40 min, which was highly specific and had no cross-reaction with other fish pathogens or other *Aeromonas* species. The detection limit was comparable to the sensitivity of the fluorescent quantitative PCR method reported in the literature, but the detection time was reduced from 180 min, which included the extraction of the bacterial genome, to 40 min. We also evaluated the applicability of this method in real-world detection, and in the detection of 74 real fish samples suspected to be infected with hemorrhagic lesions, there was good diagnostic agreement between the LAMP method and the real-time qPCR method reported in the literature (Figure 5).

When actually testing fish samples in clinical practice, the content of *Aeromonas hydrophila* in the samples is not only small, but also usually affected by disinfection, breeding environment, and radiation, and it is often in a damaged state. In addition, the fish matrix with complex components will also affect the detection sensitivity. In order to better reproduce the rapid detection process of actual samples, this study used DNA lysis reagents to obtain bacterial genomic DNA crude extraction products as templates for LAMP testing and explored its sensitivity under simple DNA lysis and extraction conditions. According to reports, using the LAMP detection reagent in this paper, the sensitivity of the LAMP reaction using the purified bacterial genomic DNA as a template is 10 pg. In this paper, the sensitivity of LAMP using the crude nucleic acid extract as a template is 559 pg, which reflects the rapid detection level of actual samples. Our newly developed LAMP method has specificity and sensitivity. After optimizing the reaction time and temperature, and following a simple DNA cleavage process, the detection of the tested sample can be completed in 40 min, meeting the requirements of rapid detection. Moreover, the isothermal amplification technology does not need to set thermal cycle steps at different temperatures, such as denaturation, annealing, and extension of PCR amplification; it shortens the reaction time; and is more suitable for instant detection in breeding sites and non-laboratory environments, which has potential advantages. It can be used for large-scale on-site investigation and on-site breeding screening of *Aeromonas hydrophila* infection in order to achieve early and rapid diagnosis.

## Figures and Tables

**Table 1:** Primers and probes for LAMP and qPCR methods.

Method	Primer Probe (Site)	Nucleic Acid Sequences (5′ → 3′)	Source
LAMP	F3(516–534 bp)	ACTTGTTCTTGGTGGTCAC	This research
	B3	CAACGACAGCGACACC	
	FIP(F1c + F2) (646–663 bp + 571–588 bp)	CTGGTCAAGACGGTGGTGGTTGGTGGCGGTATCGTA	
	BIP(B1c + B2) (507–529 bp + 572–588 bp)	GCCACTTGAACTTGTTCTTGGTGTACGATACCGCCACCAA	
	LoopF	TCAACGACAGCGACACC	
	LoopB(531–548 bp)	GTCACCTTCTCGCTCAGG	
qPCR	FW	ACC GCA AGA TCA ACG A TA CCG AGT	RONG W, et al. [14]
	RV	ATC CAG CGA GAT CCG CAC TAT CTT	
	Probe	FAM-AAC ATC TCG CTG GTT TAC CGG GTC AA-TAMRA	

## Data Availability

The data presented in this study are available on request from the corresponding author. The data are not publicly available due to the details of the data from which we report our results are stored in the instrumentation facility and there is no public release of links to archived datasets analyzed or generated during the study, as there are currently no relevant platforms for data release.
